# Prediction and detection models for acute kidney injury in hospitalized older adults

**DOI:** 10.1186/s12911-016-0277-4

**Published:** 2016-03-29

**Authors:** Rohit J. Kate, Ruth M. Perez, Debesh Mazumdar, Kalyan S. Pasupathy, Vani Nilakantan

**Affiliations:** Department of Health Informatics and Administration, University of Wisconsin-Milwaukee, Milwaukee, WI 53211 USA; Patient Centered Research, Aurora Research Institute, Aurora Health Care, Milwaukee, WI 53233 USA; Milwaukee Kidney Associates, Milwaukee, WI 53212 USA; Division of Health Care Policy & Research, Robert D. and Patricia E. Kern Center for the Science of Health Care Delivery, Mayo Clinic, Rochester, MN 55902 USA

**Keywords:** Acute kidney injury (AKI), Prediction, Detection, Machine learning, Modeling, Elderly

## Abstract

**Background:**

Acute Kidney Injury (AKI) occurs in at least 5 % of hospitalized patients and can result in 40–70 % morbidity and mortality. Even following recovery, many subjects may experience progressive deterioration of renal function. The heterogeneous etiology and pathophysiology of AKI complicates its diagnosis and medical management and can add to poor patient outcomes and incur substantial hospital costs. AKI is predictable and may be avoidable if early risk factors are identified and utilized in the clinical setting. Timely detection of undiagnosed AKI in hospitalized patients can also lead to better disease management.

**Methods:**

Data from 25,521 hospital stays in one calendar year of patients 60 years and older was collected from a large health care system. Four machine learning models (logistic regression, support vector machines, decision trees and naïve Bayes) along with their ensemble were tested for AKI prediction and detection tasks. Patient demographics, laboratory tests, medications and comorbid conditions were used as the predictor variables. The models were compared using the area under ROC curve (AUC) evaluation metric.

**Results:**

Logistic regression performed the best for AKI detection (AUC 0.743) and was a close second to the ensemble for AKI prediction (AUC ensemble: 0.664, AUC logistic regression: 0.660). History of prior AKI, use of combination drugs such as ACE inhibitors, NSAIDS and diuretics, and presence of comorbid conditions such as respiratory failure were found significant for both AKI detection and risk prediction.

**Conclusions:**

The machine learning models performed fairly well on both predicting AKI and detecting undiagnosed AKI. To the best of our knowledge, this is the first study examining the difference between prediction and detection of AKI. The distinction has clinical relevance, and can help providers either identify at risk subjects and implement preventative strategies or manage their treatment depending on whether AKI is predicted or detected.

## Background

Acute Kidney Injury (AKI) is a common clinical event among hospitalized patients, affecting at least 5 % of patients admitted to hospitals and over 25 % of patients in the intensive care unit. AKI results in significant morbidity and mortality (as high as 40–70 %), and even following resolution, can lead to severe renal impairment progressing to dialysis dependency, resulting in prolonged hospital stays and associated health costs [1, 2]. AKI occurs over the course of a few hours to days and is potentially reversible if detected and managed early in the course of the condition. Over the past few decades, AKI rates in older adults have been steadily increasing due to multiple contributing factors, such as aggressive surgical and medical treatments; increasing numbers of chronic and comorbid illnesses; greater use of nephrotoxic medications and imaging agents; and longer exposures to chronic diseases and nephrotoxins [1, 3]. Older persons who develop AKI also have higher rates of short- and long-term mortality; subsequent chronic kidney disease (CKD), including end-stage renal disease (ESRD); prolonged hospital stays; transitions to sub-acute care facilities; AKI-related morbidity; functional decline and related health care costs.

The heterogeneous etiology and pathophysiology of AKI complicates its diagnosis and medical management. Due to this, a national consortium was formed to develop consensus based guidelines (RIFLE criteria) for AKI diagnosis [4], followed by further refinement with the AKIN criteria [5]. However, reliance on a rise in serum creatinine alone as the gold standard for diagnosis is problematic in older adults because of age-related declines in glomerular filtration rates, which can affect baseline serum creatinine, and because serum creatinine is influenced by muscle mass, nutritional status and volume distribution. Although decreased urine output is associated with early course of AKI, this value is frequently not monitored and missed till AKI is established.

Despite these drawbacks, it is important to note that 20–30 % of AKI in the hospital setting is predictable and avoidable if all risk factors are identified, quantified and utilized in the clinical setting for risk profiling of patients. Recently, the ASSESS-AKI study [6] was established to examine how AKI can predict risk of developing CKD, cardiovascular events and death, but AKI risk prediction itself was not addressed in this study. Also important to note is the difficulty in predicting risk of AKI in older adults, as many variables used for risk prediction become less predictive as age increase [7].

In this study, we built machine learning models to predict at 24 h of admission whether a patient will develop AKI during the rest of the hospital stay, and to detect AKI (anytime during hospital stay) for hospitalized patients over the age of 60. To the best of our knowledge, there are no studies in the literature examining the difference in prediction vs. detection of AKI–the distinction between the two could be clinically very important as it can help providers either plan preventive care or manage treatment/plan depending on whether AKI is predicted or detected. We tested four separate and different types of models and their ensemble to allow us to compare and contrast different methods for prediction and detection of AKI.

## Methods

### Data collection

Patients older than 60 years of age with at least a one day hospital stay (encounter) in 2013 at any of Aurora Health Care’s (15) hospitals formed our retrospective cohort (*n* = 32,076). All these hospitals use the same electronic health record (EHR), follow standardized order sets, and are located in the southeastern region of the Wisconsin state. Aurora Health Care’s EHR system was queried to obtain structured data corresponding to the patient cohort. The structured data included demographic information, admission and discharge dates and times, surgeries, comorbidities, family history, medications and laboratory values. Each piece of this structured data also had a timestamp corresponding to when it was recorded. Using this information and the timestamps on the structured data, it was possible to appropriately associate patients’ structured data with their encounter. This study was approved by the Institutional Review Board at Aurora Health Care.

### AKI definition

AKI was defined using the AKIN criteria [5] using any two serum creatinine measurements taken within 48 h of each other during an encounter. Given that a patient may have AKI during one encounter and not during another, in this study, encounters rather than patients were classified as ‘with AKI’ or ‘without AKI’. Out of total 25,521 encounters that were included in our data, patients acquired AKI during 2,258 (8.84 %) encounters.

### Study design

Two separate tasks were considered: *predicting* whether a patient will acquire AKI during their encounter and *detecting* if a patient has acquired AKI sometime during their encounter that would otherwise go undetected. While predicting AKI is important to enable better preventive care, detecting undiagnosed AKI is also important to enable an alert system that will lead to suitable treatment measures.

### Predicting AKI

AKI prediction models were built using machine learning methods to predict at 24 h from admission whether a patient will develop AKI later during the hospital stay. Positive examples were those in which AKI was acquired after 24 h (1,782) and negative examples were the encounters during which AKI was never acquired (23,263). There were no encounters shorter than 24 h in our data. Encounters during which AKI was acquired within 24 h of admission were not used as examples because the model is being trained to predict AKI at 24 h from admission.

Demographic information, comorbidities, family history, medications and laboratory values extracted from the structured part of EHRs were used as predictive variables by the models. For each of these variables, only the last recorded value before 24 h after admission was used for each example. If no such value existed for a hospital stay then its value was taken as “unknown.” Serum creatinine was not used as a predictive variable as it was used to determine “gold-standard” positive and negative examples. Comorbidity and medication variables took either “yes” or “no” values. If a patient had a comorbid condition or was prescribed a medication anytime in the past then its value was considered to be “yes” because the patient would be susceptible to AKI. The family history parameter was “yes” only if the corresponding field in the EHR mentioned kidney or a kidney related disease. For every laboratory value variable only the last value recorded within 24 h from admission was used. Unlike medications or comorbidities, a laboratory value prior to the encounter was not used.

### Detecting AKI

For AKI detection, positive examples were encounters during which AKI was acquired (2,258) and negative examples were those during which AKI was never acquired (23,263). Unlike the AKI prediction which had a fixed time of prediction at 24 h from admission, this task did not have a fixed time of detection since AKI could be acquired anytime during the encounter and the model needs to detect whenever it happens. However, the positive and negative examples require timestamps to represent the temporal clinical scenario for applying the model. Positive examples used the time AKI was acquired (as determined by the timestamp of the second serum creatinine measurement which met the AKIN criteria) as its timestamp: this is when the model will be expected to detect AKI. For negative examples, any time during the encounter could be used as timestamps because at any of these times AKI was not present. To limit one timestamp per negative example, we chose to use the timestamp of the last serum creatinine measurement taken during the encounter because it signifies that the patient was still prone to developing AKI. We avoided using any fixed timestamp, say discharge time, for negative examples because instead of learning to detect AKI, the model may simply learn to distinguish between patients about to be discharged and patients who are to continue their stay.

As in the task of predicting AKI, the demographic information, comorbidities, family history, medications and laboratory values were used as predictive variables in the same manner. For each of these variables, only the last recorded value before the example’s timestamp was used otherwise the value was taken as “unknown.” This was the reason associating timestamps with the examples was necessary.

### Experimental methodology

Four different machine learning methods - Logistic Regression [8], Support Vector Machines (SVMs) [9], Decision Trees [10], Naïve Bayes [11] as well as an ensemble [12] of all these methods, were used for building our models using the freely and publicly available Weka software [13]. We chose these four methods because they are well-known and represent different types of machine learning methods. Logistic regression and SVMs are statistical methods, decision tree is a rule-based method, and naïve Bayes is a probability-based method. Decision tree models are human-interpretable; logistic regression models are informative as they show relation between predictor variables and dependent variable in terms of odds ratios; but SVMs and naïve Bayes models are not very human-interpretable. However, SVMs have been theoretically as well as experimentally shown to work well even on prediction tasks involving thousands of variables; and naïve Bayes, in spite of its naïve assumption, performs competitively on many real-world tasks and is also extremely fast to train and test. Weka’s implementations of these methods also have default mechanisms for handling unknown values of variables in the data which we used for our experiments.

An ensemble method combines multiple classification methods and typically obtains better results than the component classifiers [12]. However, it may not improve results if the component classifiers lack in diversity and agree most of the time in their output classifications. There are several methods to build ensembles, we used the stacking method available in Weka in which the outputs of the component classifiers are used as variables by a top-level classifier which is also trained using the training data.

All models were evaluated using the standard ten-fold cross-validation [14]. In this procedure, the entire data is first randomly divided into ten equal parts, the models are trained on nine parts and tested on the tenth part, and this process is repeated ten times each time using a different part for testing. The results of all these ten folds are then combined to compute the evaluation scores. All the machine learning methods we used are capable of giving a confidence score with their output AKI/Non-AKI classification. By varying the threshold on this confidence score, one can trade-off between true positive rate (sensitivity) with false positive rate (1-specificity) and thus generate an entire receiver operating characteristic (ROC) curve. We used the standard measure of area under ROC curve (AUC) to report and compare performance of the models.

Our datasets for both the prediction and detection tasks were highly unbalanced with the number of negative examples (non-AKI) more than thirteen times the number of positive examples (AKI) for the prediction task and more than ten times for the detection task. With such an unbalanced data, a machine learning model set to optimize evaluation scores may do so simply by calling all test examples negative (this way it will have, for example, more than 90 % of the test examples correctly classified on the detection task). But such a model will be practically of little use. In order to make the model optimize its performance on both the classes, a weight is assigned to the minority class based on how damaging it will be to misclassify it compared to misclassifying the majority class. In Weka, weight for a class can be specified by using its cost-sensitive meta-classifier. In order to determine the right weight of the positive class, in each fold we did internal ten-fold cross-validations within its training data with different weights. The weight that gave the maximum AUC on internal cross-validation was then used to build the model using the entire training data for that fold. We varied the weight from 1, 2, 4, 6, …, 18, 20 for internal cross-validations for each of the machine learning methods we used.

We used Weka’s “SMO” method for SVM, “J48” method for decision trees, “Logistic” method for logistic regression and “NaiveBayes” method for naïve Bayes. Decision tree’s “minimum number of instances per leaf” parameter was also set through internal cross-validation (together with the weight of the positive class) out of 20, 40, 60, …, 280, 300. We found that it was computationally impractical to determine the best values of SVMs’ noise and kernel parameters through internal cross-validation because of the long computational times. Through pilot experiments we found that Weka’s default parameter setting for SVMs (noise parameter = 1 and linear kernel) to be the best for our dataset and used these values. We, however, determined the best value of the weight of the positive class for SVMs through internal cross-validation. Logistic regression and naïve Bayes methods did not have important parameters to set besides the weight of the positive class.

An ablation study was performed to determine the relative contributions of comorbidity, medications and laboratory values variables. Learning curves were also plotted to see how the performance changes with increasing number of training examples.

## Results

### Patient characteristics

**Fig. 1:**
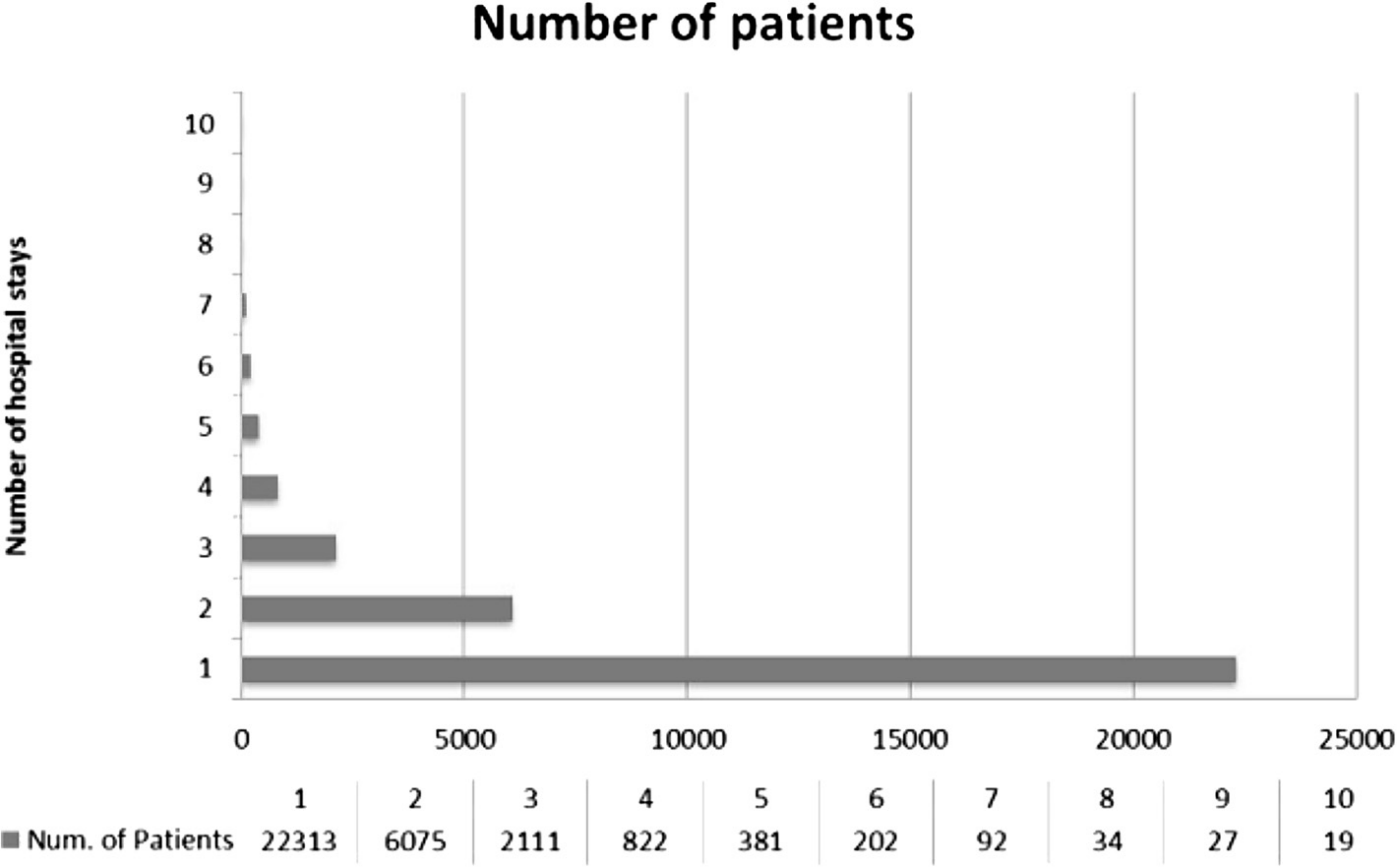
Number of hospital stays of the 32,076 hospitalized patients in the year 2013

**Fig. 2:**
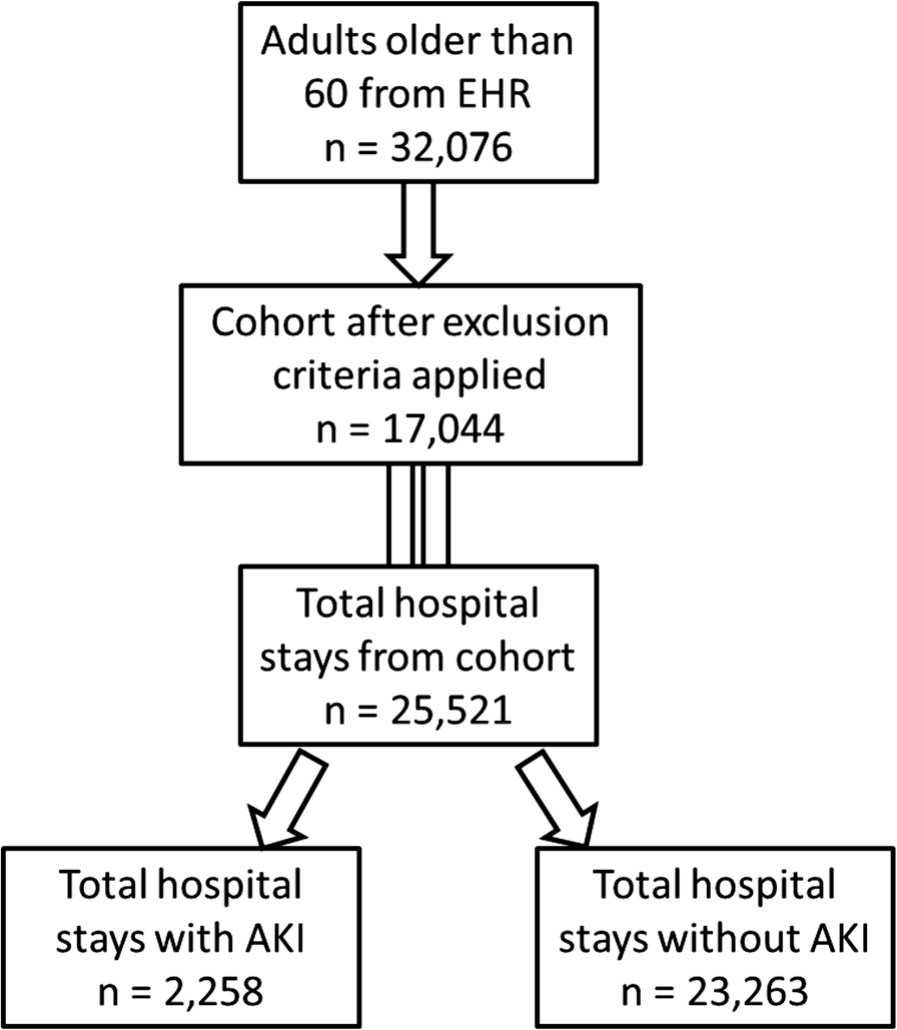
Flowchart depicting number of patients that were included in analysis after exclusion criteria. The total included encounters were divided as AKI or without AKI

Figure 1 shows the number of encounters (hospital stays) in the retrospective cohort of patients for the time period of the study. A majority of patients had a single (*n* = 22,313) or two encounters (*n* = 6,075) with only 374 patients having 6 or more encounters. Figure 2 shows the number of patients included in analysis after exclusion criteria were applied. Patients with chronic kidney disease stages III, IV and V, organ transplant recipients, and those with less than two serum creatinine measurements were excluded with remaining 25,251 encounters used for analysis. The total included encounters were divided as AKI (*n* = 2,258) or without AKI (*n* = 23,263).

Patient characteristics separated by AKI and non-AKI status are depicted in Table 1. All demographics except for age, those belonging to a race other than Black or White and family history of kidney disease were statistically different between AKI and non-AKI encounters. We note that some categorical values do not add to 100 % in Table 1 because of some missing or unknown values in the data. The majority of laboratory values, medications and comorbidities had significant differences between the two groups, with the exception of platelet count, temperature, NSAID, Cisplatin and acyclovir use, disorders of lipoid metabolism and rhabdomyolysis.

**Table 1:** Distribution of various variables in AKI and non-AKI encounters. For numeric variables, mean and standard deviation are shown; categorical variables are shown with the number of occurrences and percentages

Variable	AKI (2258)	Non-AKI (23263)	*P*-value
Demographics			
Age	75.2 ± 9.5	75.3 ± 9.6	0.799
BMI	29.7 ± 8.2	28.4 ± 7.3	**<0.0001**
Race = White	1974 (87.4 %)	20940 (90.0 %)	**<0.0001**
Race = Black	198 (8.8 %)	1571 (6.8 %)	**<0.0004**
Race = Other	27 (1.2 %)	240 (1.0 %)	0.5331
Sex = Female	1101 (48.8 %)	12426 (53.4 %)	**<0.0001**
Sex = Male	1154 (51.1 %)	10791 (46.4 %)	**<0.0001**
Tobacco Use = Never	767 (34.0 %)	8601 (37.0 %)	**<0.005**
Tobacco Use = Quit	1281 (56.7 %)	12118 (52.1 %)	**<0.0001**
Tobacco Use = Yes	193 (8.5 %)	2360 (10.1 %)	**<0.02**
Alcohol Use	705 (31.2 %)	8037 (34.6 %)	**<0.002**
Family History	37 (1.64 %)	442 (1.90 %)	0.4280
Laboratory Values			
BUN	25.81 ± 14.27	17.45 ± 8.63	**<0.0001**
AST	35.49 ± 29.02	27.79 ± 18.7	**<0.0001**
Troponin	3.98 ± 3.13	1.31 ± 1.12	**<0.0001**
Blood Bilirubin	0.77 ± 0.56	0.60 ± 0.37	**<0.0001**
Platelet Count	214.33 ± 92.98	215.11 ± 85.71	0.686
Heart Rate	84.2 ± 19.0	81.3 ± 18.7	**<0.0001**
Temperature	98.2 ± 1.2	98.1 ± 1.6	0.08
BP systolic	140 ± 32	138 ± 28	**<0.01**
BP diastolic	72 ± 16	71 ± 14	**<0.04**
Medications			
ACE Inhibitors	1219 (54.0 %)	10869 (46.7 %)	**<0.0001**
ARB	422 (18.7 %)	3851 (16.6 %)	**<0.02**
NSAIDS	821 (36.36 %)	8619 (37.05 %)	0.5312
Lipid Lowering Drugs	1539 (68.16 %)	15233 (65.48 %)	**<0.02**
Diuretics	1750 (77.50 %)	13654 (58.69 %)	**<0.0001**
ACE Inhibitors or NSAIDS or Diuretics	2052 (90.88 %)	19111 (82.15 %)	**<0.0001**
ARB or ACE Inhibitors or NSAIDS or Diuretics	2076 (91.94 %)	19580 (84.17 %)	**<0.0001**
K Sparing	284 (12.58 %)	1786 (7.68 %)	**<0.0001**
Aminoglycoside Antibiotics	241 (10.67 %)	1986 (8.54 %)	**<0.001**
Radiocontrast Dyes	1536 (68.02 %)	14229 (61.17 %)	**<0.0001**
Cisplatin	31 (1.37 %)	267 (1.15 %)	0.3963
Acyclovir	119 (5.27 %)	1170 (5.03 %)	0.6539
Comorbidities			
Prior AKI	378 (16.74 %)	1470 (6.32 %)	**<0.0001**
Diabetes	223 (9.88 %)	1410 (6.06 %)	**<0.0001**
Hyperlipidemia	392 (17.36 %)	3127 (13.44 %)	**<0.0001**
Hypercalcemia	32 (1.417 %)	222 (0.95 %)	**<0.05**
Thrombocytopenia	103 (4.56 %)	567 (2.43 %)	**<0.0001**
Hypertension	453 (20.06 %)	3518 (15.12 %)	**<0.0001**
Heart Failure	272 (12.05 %)	1442 (6.20 %)	**<0.0001**
Coronary Artery Disease	250 (11.07 %)	1670 (7.18 %)	**<0.0001**
Disorders of Lipoid Metabolism	134 (5.93 %)	1413 (6.07 %)	0.8265
Pancreatitis	51 (2.26 %)	342 (1.47 %)	**<0.005**
Rhabdomyolysis	19 (0.84 %)	164 (0.70 %)	0.5464
Congestive Heart Failure	272 (12.04 %)	1442 (6.20 %)	**<0.0001**
Sepsis	230 (10.19 %)	1324 (5.69 %)	**<0.0001**
Respiratory Failure	363 (16.08 %)	1271 (5.46 %)	**<0.0001**

The *p*-values less than 0.05 are shown in bold

### Classification results

**Fig. 3:**
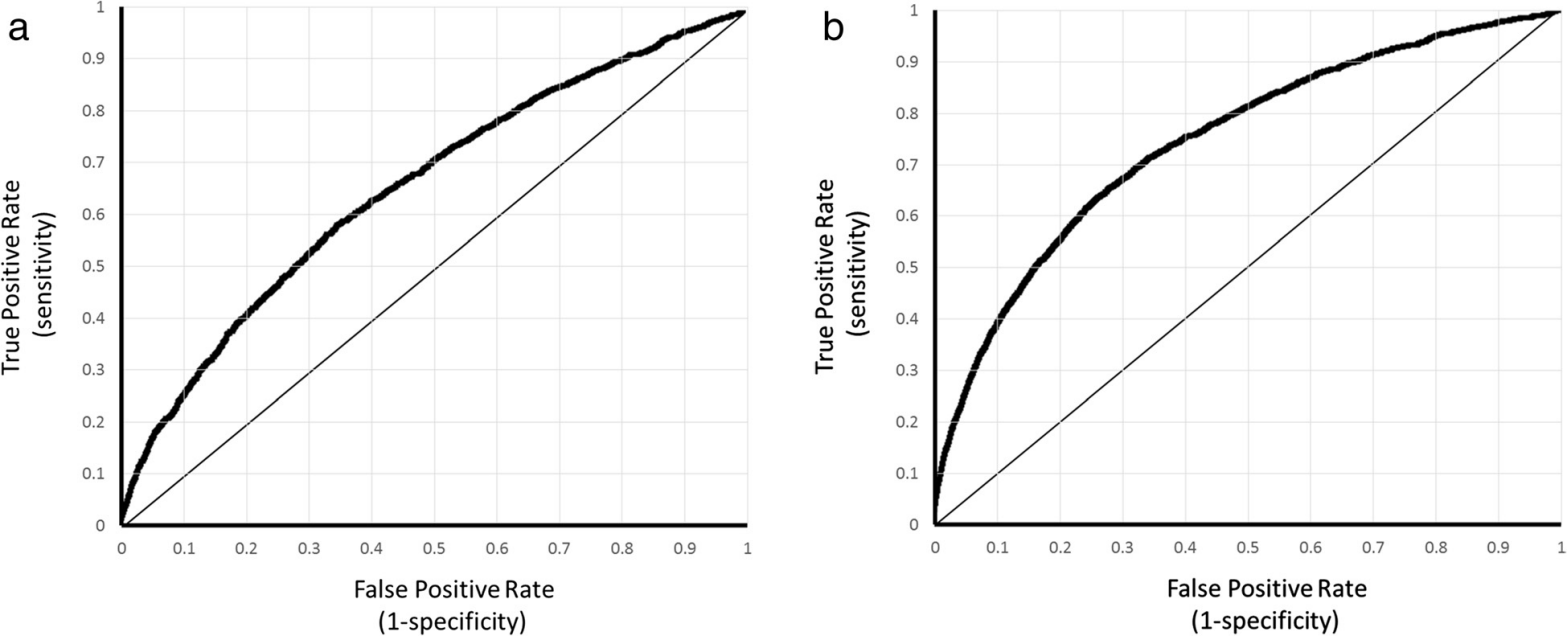
**a** ROC curve of the logistic regression model for predicting AKI. **b** ROC curve of the logistic regression model for detecting AKI

The area under ROC (AUC) ranged between 0.621 to 0.664 for predicting AKI and between 0.692 to 0.743 for detecting AKI (Table 2) between the four machine learning methods and their ensemble. The performance on the detection task was clearly better than that on the prediction task for each model tested, indicating that it is easier to detect AKI than to predict it. While all the methods obtained competitive results on both prediction and detection, logistic regression obtained the best results for AKI detection (AUC 0.743) and was a close second to the ensemble method for AKI prediction (AUC ensemble method: 0.664, AUC logistic regression: 0.660). An ensemble model typically improves over its component models if they disagree on some of their outputs in such a way that when one model incorrectly classifies an example most other models correctly classify it. Hence these results indicated that our models mostly agreed well with one another and could not correct their mistakes by forming an ensemble. Note that ROC curve for a random classifier is a diagonal line with AUC 0.5, hence our models outperform it. Figure 3a and b show the ROC curves obtained by the logistic regression model for prediction and detection respectively. The corresponding curves for other models are not shown for clarity as they are close to each other. An ROC curve shows the entire range of sensitivity and specificity obtainable by the model allowing a user to trade-off between them to choose a particular setting. For example, in Fig. 3a, one can choose 75 % sensitivity for AKI prediction and obtain 43.6 % specificity, or choose 75 % specificity and obtain 45.5 % sensitivity. One can also choose 80 % sensitivity and obtain 36.9 % specificity, or choose 80 % specificity and obtain 40.6 % sensitivity. Similarly, in Fig. 3b, one can choose 75 % sensitivity for AKI detection and obtain 61.1 % specificity, or choose 75 % specificity and obtain 62.6 % sensitivity. One can also choose 80 % sensitivity and obtain 52.7 % specificity, or choose 80 % specificity and obtain 55.7 % specificity.

**Table 2:** Area under ROC curves and 95 % confidence intervals obtained using different machine learning methods for predicting and detecting AKI during hospital stay. In each column, the highest ROC value is shown in bold and the values found statistically significantly different (*p* < 0.05; two-tailed paired *t*-test) from it are indicated with a ^a^symbol

Method	Prediction at 24 h (95 % CI)	Detection (95 % CI)
Logistic Regression	0.660 (0.647–0.673)	**0.743 (0.732–0.755)**
Naïve Bayes	^a^0.654 (0.639–0.669)	^a^0.699 (0.684–0.715)
Decision Trees	^a^0.639 (0.627–0.651)	^a^0.725 (0.717–0.733)
Support Vector Machine	^a^0.621 (0.609–0.633)	^a^0.692 (0.682–0.702)
Ensemble	**0.664 (0.651–0.676)**	^a^0.738 (0.727–0.748)

### Ablation study

In order to determine the relative contributions of the major categories of variables, we conducted an ablation study where we excluded laboratory value, medication and comorbidity variables one group at a time, and repeated the same process of training and evaluation. Table 3 shows the ablation results for the logistic regression classifier which was found to be the best classifier on our data. It is clear that the performance always dropped on both the tasks after removing any category of variables; however, it is interesting to observe how much the performance dropped in each case. The performance on detection task dropped dramatically from 0.743 AUC to 0.668 AUC on removing laboratory values which shows that they play the most significant role in helping to detect AKI. However, excluding laboratory values incurred a very small drop in performance on the prediction task (AUC 0.66 to AUC 0.656) whereas the largest drop occurred when comorbidities were removed (AUC 0.66 to AUC 0.625) which shows that they are the more predictive of AKI when compared to medications or laboratory values.

**Table 3:** Results of ablation study for area under ROC curve and 95 % confidence intervals obtained on the two tasks using the logistic regression classifier. The values found statistically significantly different (*p* < 0.05; two-tailed paired *t*-test) from the value in the “All” column in the same row are indicated with a ^a^symbol

	All (95 % CI)	Exclude Labs (95 % CI)	Exclude Meds (95 % CI)	Exclude Comorbidities (95 % CI)
Prediction at 24 h	0.660	0.656	0.647	0.625^a^
	(0.647–0.673)	(0.643–0.668)	(0.633–0.662)	(0.611–0.638)
Detection	0.743	0.668^a^	0.728^a^	0.705^a^
	(0.732–0.755)	(0.652–0.683)	(0.707–0.749)	(0.686–0.724)

### Learning curves

**Fig. 4:**
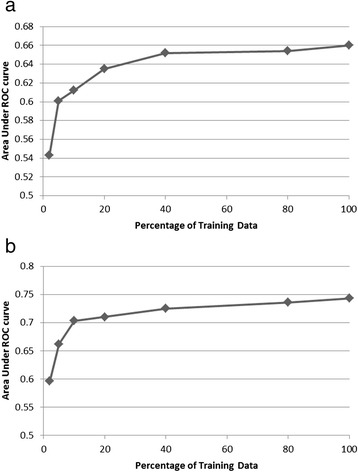
**a** Learning curve for the AKI prediction task obtained using the logistic regression model. **b** Learning curve for the AKI detection task obtained using the logistic regression model

Figure 4a and b show the learning curves obtained on the prediction and detection tasks respectively using the logistic regression classifier which performed best on this data. The corresponding curves for other models are qualitatively similar and are not shown. To obtain each point on a learning curve, the same training and evaluation procedure was employed as before but using only a portion of the entire training data in each fold. A larger portion of training data always comprised all the examples that a smaller portion of training data included. The test data, however, remained exactly the same. From the graphs one can see that the performance grows rapidly in the beginning with increasing amounts of training data and reaches very close to peak with around 40 % of the training data. The graphs also show that while the performance has mostly plateaued, it may still go up slightly with more training data.

#### Odds ratios

**Fig. 5:**
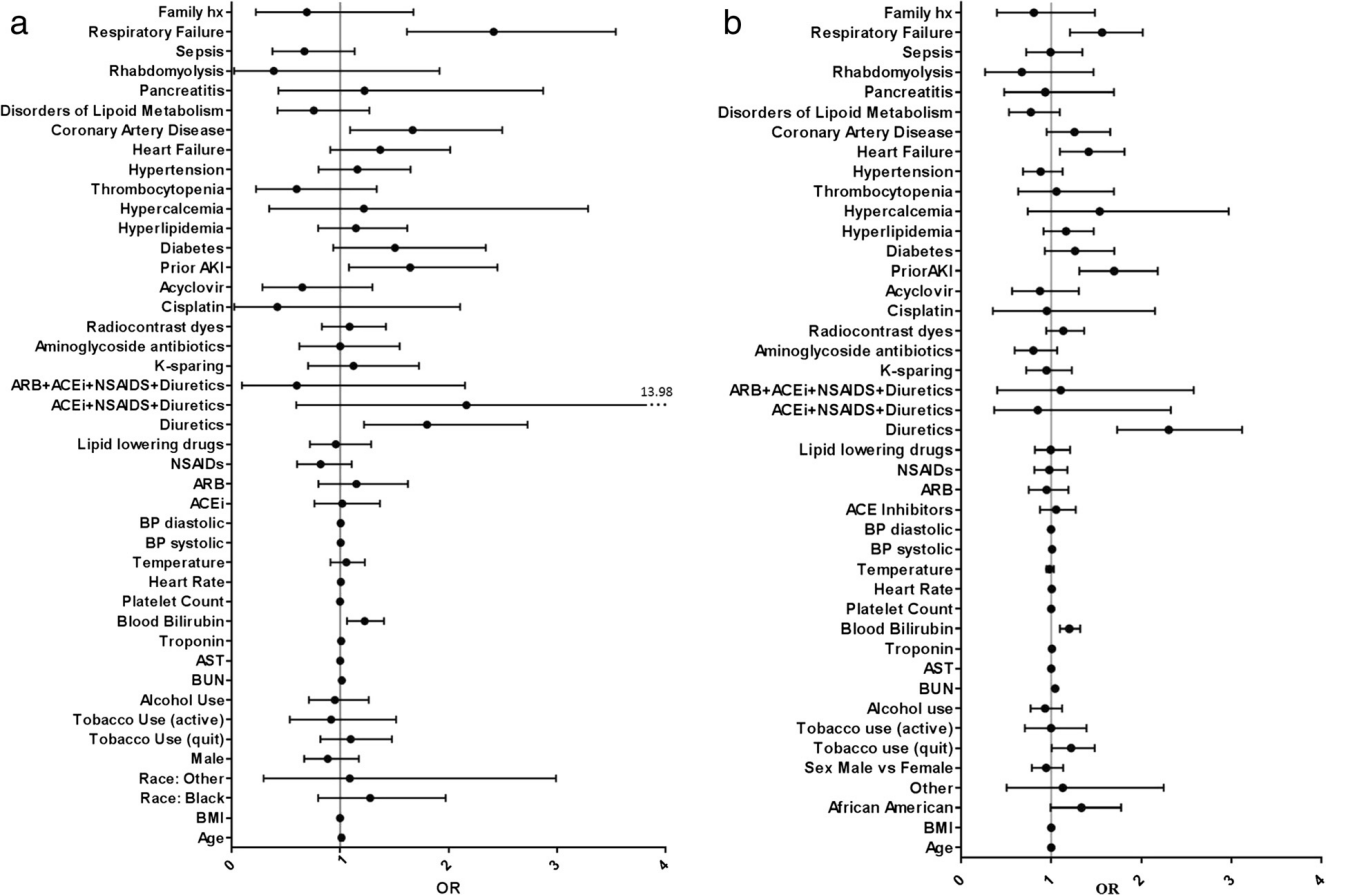
**a** Odds ratios for AKI prediction. The lines indicate 95 % confidence intervals. **b** Odds ratios for AKI detection. The lines indicate 95 % confidence intervals

Odds ratios of the variables for the prediction and detection tasks obtained using logistic regression are seen in Fig. 5a and b respectively. Weka software does not give confidence intervals on its odds ratios, hence we used R statistical software to obtain the odds ratios and their confidence intervals. Variables that had high odds ratios for AKI prediction vs. AKI detection were different –for AKI prediction: use of diuretics (OR 1.801) or combination drugs such as NSAIDs, ACE inhibitors and diuretics (OR 2.165), history of prior AKI (OR 1.646), comorbidities such as coronary artery disease (OR 1.669), diabetes (OR 1.505) and respiratory failure (OR 2.415) all had strong association with AKI. On the detection task, use of diuretics (OR 2.305), history of prior AKI (1.697), presence of comorbid conditions such as hypercalcemia (OR 1.538) heart failure (OR 1.415) and respiratory failure (OR 1.564) each had strong association with the AKI class.

## Discussion

### AKI detection vs. AKI prediction

Each modeling method used to predict and detect AKI performed fairly well although logistic regression was the best. The choice between developing an AKI prediction or an AKI detection model is ultimately dependent on whether the model will be used in clinical decision support tools for prevention of AKI or for management of the disease in the hospital setting.

A few computer based algorithms and predictive models for AKI risk have been used in post-operative complications and ICUs [4, 15–19], but a clear linkage of risk factors for AKI is not evident in the current literature. Recently, the ASSESS AKI study examined how AKI can predict risk of developing CKD, cardiovascular events and death, but AKI risk prediction itself was not addressed [6]. Other studies have developed risk stratification models for AKI using structured EHR data [7, 20]. However, they did not specify the time point at which AKI is predicted. This is an important distinction from our study because it can have an impact on patient care and management of the disease. After a patient is admitted to hospital, the sooner AKI is predicted the higher the opportunity to prevent established AKI and its associated morbidity and cost. However, the variables used to predict AKI will be better indicative of AKI if their values are recorded closer to the time AKI is actually acquired. On the other hand, if the prediction is made too late, a patient may already acquire AKI before the prediction time thus making the prediction pointless. Hence there is a trade-off between how soon to predict AKI and the accuracy of predicting AKI.

Table 4 depicts in how many hospital stays AKI was acquired within different time intervals from admission (as determined by the timestamp of the second serum creatinine measurement which met the AKIN criteria). It is evident that the number of AKI acquired cases increase with the time from admission; and about half of these cases happen by 48 h. In contrast to the earlier studies [7, 20] where researchers did not make it clear exactly when, from the time of admission, the models will be applied to make AKI prediction, in the current study, our models make AKI prediction at 24 h from admission as a reasonable compromise for the trade-off between the models’ usefulness (clinical relevance) and its predictive power (scientific rigor). If needed, our models can be easily adapted to make predictions at any other desirable time from admission (12 h, 48 h etc.).

**Table 4:** Number of encounters in which AKI was acquired within different intervals from time of admission

Within time from admission (hours)	Number of AKI cases
6	8
12	92
24	476
36	725
48	1166
60	1313
120	1796
240	2103
Through end of stay (total)	2258

Aside from predicting AKI early in the encounter, it is equally important to detect AKI any time throughout the encounter to prevent AKI from going undiagnosed. A patient may acquire AKI during an encounter while the providers are focused on treating other illnesses. In such situations the model will detect AKI and alert the providers. Different than the model posed by Wilson et al. [21] in which their alert system was based on a change in serum creatinine, our model is capable of detecting AKI in the absence of serum creatinine measurement because once this measurement is taken, one can reliably know whether AKI is present or not without needing the model. Thus our model’s alert may, in fact, prompt the providers to take serum creatinine measurement which they might have overlooked.

### Factors affecting AKI prediction vs. AKI detection

We further explored the factors and variables important in either prediction or detection of AKI. The logistic regression model was used to obtain odds ratios for all the variables. Of all the variables, only a few stood out for prediction of AKI (use of combination drugs, history of prior AKI and comorbidities such as coronary artery disease and respiratory failure). In our analysis, although independent use of ACE inhibitors and NSAIDs did not increase AKI prediction, combining these drugs with diuretics increased the odds ratio significantly, with diuretics being the single largest contributor to the prediction. This finding is also consistent with a study in which the combination of these drugs was found to increase the risk of AKI [22]. Diabetes, coronary artery disease and respiratory failure all contributed to the higher AKI risk prediction. The link for coronary artery disease and AKI is justifiable as previous reports have shown that AKI risk increases after cardiac surgical procedures especially cardiac bypass graft surgery [23, 24]. Our current results for respiratory failure as a good indicator for AKI risk prediction is also similar to a recent report indicating that respiratory distress syndrome is independently associated with AKI and may promote AKI [25]. Another large study (n ~ 2 million patients) was conducted to quantify the risk of AKI associated with Type 2 diabetes using the General Practice Research Database in the UK_ENREF_24 [26]. The researchers concluded that even after adjustment in known risk factors, elderly patients with Type 2 diabetes have an increased risk of AKI. Our study results corroborate well with these previously published reports.

Similar to the AKI prediction, a few variables stood out as being important factors for AKI detection. History of prior AKI, use of diuretics and presence of comorbid conditions such as respiratory failure were significant for both AKI risk prediction as well as AKI detection. Presumably in critically ill patients, the incidence of sepsis, respiratory failure, etc. are more prevalent leading to higher incidence of AKI in this population. Detection of AKI (which might otherwise go undiagnosed) is nevertheless very important as it can help guide management of the AKI in terms of volume resuscitation and hydration to prevent any permanent damage to the kidneys and dependence on renal replacement therapy.

### Limitations of study

The results of this study should be interpreted with respect to several limitations. First, our model was based off an older cohort (60 years and older) which may limit the generalizability to a wider age group. Second, we limited inclusion to those presenting with chronic kidney disease I and II. This was done to achieve a more homogeneous dataset with which to train our model. However, those with chronic kidney disease III, IV and V are at an increased risk for developing AKI and accurate detection/prediction of this is currently not in the scope of our model. Third, any variable which was missing for more than 20 % of the population was not included in our analysis. This might have limited our model’s performance. Finally, the models that we used relied solely on structured information found in the electronic health record.

Future studies will focus on using machine learning and natural language processing techniques to extract AKI-relevant information from unstructured clinical notes. It is expected that the information held within the clinical notes will improve the predictive models.

## Conclusions

This study was first to consider two distinct tasks of predicting and detecting AKI in hospitalized older adults. Predictive models were built for both the tasks using different machine learning methods trained on large patient data of hospital stays. All models performed well on both the tasks but were better at detecting AKI than predicting AKI. Through ablation study, laboratory values were found to be most important for detecting AKI while comorbidities were found to be most important for predicting AKI. Among the models, logistic regression performed the best. This model found certain comorbidities and drug combinations to be particularly good predictors of AKI which also has support in literature. The models developed in this study could help in early identification of subjects at risk as well as in early detection of AKI and allow implementation of preventative intervention in the care and management of these patients during the course of their hospital stay.

### Data availability

Our dataset consists of hospital patient data provided by Aurora Health Care, Inc. and can only be shared with its written consent.

## Abbreviations

ACE: angiotensin-converting enzyme
AKI: acute kidney injury
AUC: area under curve
NSAIDs: nonsteroidal anti-inflammatory drugs
ROC: receiver operating characteristics
SVMs: support vector machines
